# Skin and Genito-Urinary Diseases

**Published:** 1893-04

**Authors:** 


### SKIN AND GENITO-URINARY DISEASES.

---

EDITED BY DR. M. B. HUTCHINS.

---

### PECULIAR CASE OF PROSTATIC CONCRETIONS, WITH SPECIMENS.

At the October 13, 1892, meeting of the section of the New York Academy of Medicine on Genito-Urinary Surgery, Dr. E. L. Keyes presented a large number of prostatic concretions, like millet-seeds, which he had removed from a man 45 years of age. The man suffered from constant dribbling of the urine, and the urethra was always in a purulent condition. At night he suffered less than by day. He was made to pass his urine in two parts, and both parts were found to be purulent. A catheter was then introduced into the bladder, and through this perfectly clear urine flowed; the residual urine was also clear. This located the pus in the prostate. Dr. Keyes said he did not search the man for stone, as he had no symptoms of it. A central perineal incision was made and the finger introduced, and an abscess cavity in the prostate discovered. To the finger it felt like a rotten raspberry, and this seedy material was scraped out with the finger nail. The opening of the abscess was enlarged, and the entire cavity thoroughly scraped. The perineal opening was allowed to close after three weeks. There is now no more residual urine, and no dribbling from the urethra, and the man will no doubt get entirely well. He may have some trouble, however, owing to the fact that the anterior portion of his prostate was destroyed, although the mucous membrane was left.—*Journal Cutaneous and Genito-Urinary Diseases, January*, 1893.

---

### HÆMOGLOBINURIA FOLLOWING THE ADMINISTRATION OF QUININE.

Hæmoglobinuria consequent upon the exhibition of quinine is often the manifestation of a predisposition, occasionally running through families, following the administration of the drug more or less rapidly and showing a variable degree of intensity with the quantity of the dose and the susceptibility of the individual. Any of the salts of quinia, given by mouth or hypodermatically, may bring on the attack. The prognosis is good in every case, becoming grave only when the kidneys are unable to eliminate the hæmoglobin and jaundice results. The destruction of red corpuscles, indicated by the presence of hæmoglobin in the urine, exists, as the author states with positiveness from his investigations, as an initial condition in the loss of alkalinity in the blood. Therefore, it is his practice, to administer a large dose of sodium bicarbonate before quinine, in the predisposed. As a remedy for the hæmatogenous jaundice, he recommends the use of the essence of terebenthine, a practice in vogue in South America.—*Journal Cutaneous and Genito-Urinary Diseases, January*, 1893.

---

### TREATMENT OF GONORRHŒA.

Dr. W. S. James, of Cleveland, O., writes us that he has employed the following injection with excellent results in the treatment of a case of chronic gonorrhœa, where solutions of sulphate of zinc, nitrate of silver and bichloride of mercury proved inefficient, and that he has derived equal benefit from it in acute cases:

℞. Boracic acid, ʒ iss.

Tincture of iodine, ʒ ii.

Glycerine, ℥ i.

Distilled water q. s. ad., ℥ iv.

M. Sig.—To be used as an injection morning and night.

He would like other physicians to give this formula a trial.—*International Journal of Surgery.*

---

### FOR WARTY VEGETATIONS OF THE EXTERNAL GENITAL ORGANS.

The *Bulletin Gen. de Therapeutique* recommends the following formula for warty vegetations of the external genital organs :

℞. Salicylic acid, 2 gms.
Acetic acid, 30 gms.

Mix. External use.

Apply to the vegetations once or twice in twenty-four hours, by means of a small brush, two or three applications being sufficient. The pain is slight and transient, which is an advantage.—*Cin. Lancet-Clinic.*

